# Inter-study repeatability of self-gated CMR perfusion: A comparison of Fermi and compartment models

**DOI:** 10.1186/1532-429X-18-S1-P223

**Published:** 2016-01-27

**Authors:** Devavrat Likhite, Promporn Suksaranjit, Ganesh Adluru, Christopher J McGann, Brent D Wilson, Edward V DiBella

**Affiliations:** 1 Department of Radiology, UCAIR, University of Utah, Salt Lake City, UT USA; 2 Division of Cardiovascular Medicine, University of Utah, Salt Lake City, UT USA

## Background

Recent developments in cardiovascular magnetic resonance (CMR) perfusion have made it possible to rapidly acquire multiple slices continuously without the need for any ECG-triggering. Promising results have been shown for visual assessment and quantification of perfusion using self-gated techniques [1-3]. This work compares the repeatability of a free breathing ungated acquisition using the Fermi model and a compartment model.

## Methods

10 subjects were each scanned on two separate days with 9.5 ± 4.5 days between scans. Perfusion data was acquired with ECG-triggering turned off, using a radial saturation recovery turboFLASH sequence on a Siemens 3T Verio scanner. A set of four slices was acquired after a single saturation pulse. The scan parameters were 24 rays per image, TR=2.2 ms, TE=1.2 ms, 1.8 × 1.8 × 8 mm^3^ voxels. Gadoteridol 0.05 mmol/kg was injected and ~240 frames were acquired. 20 ± 5 minutes later, regadenoson was injected to induce hyperemia. The same scan protocol was followed to acquire 4 matching slices at stress. The data was reconstructed offline using a spatio-temporally constrained reconstruction technique based on [4].

The images were "self-gated" to near-systole as in [2] and used to quantify myocardial blood flow. The most basal slice with lowest SRT was selected for the AIF and the remaining three slices were used to obtain the tissue curves. The AIF and tissue curves were converted to gadolinium concentration. The tissue curves and the AIF were then fit to a Fermi model and a compartment model. Myocardial perfusion reserve (MPR) was reported as the ratio of flow values at stress and rest. To compare repeatability, a paired t-test was used to assess the difference between scans, for the two models.

## Results

**Figure 1:**
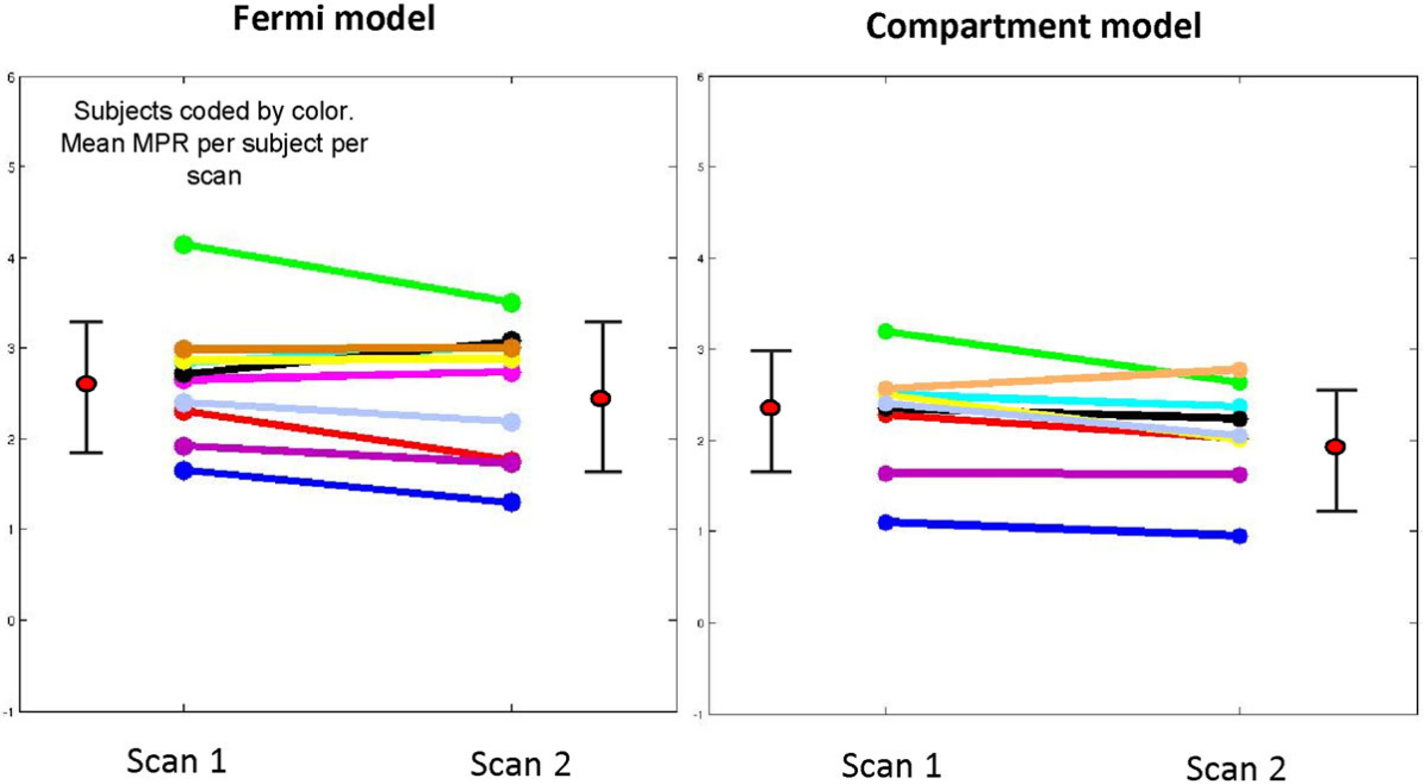
**A plot comparing the mean MPR between scan 1 and scan 2 using Fermi and compartment model using self-gated systole dataset**.

**Figure 2:**
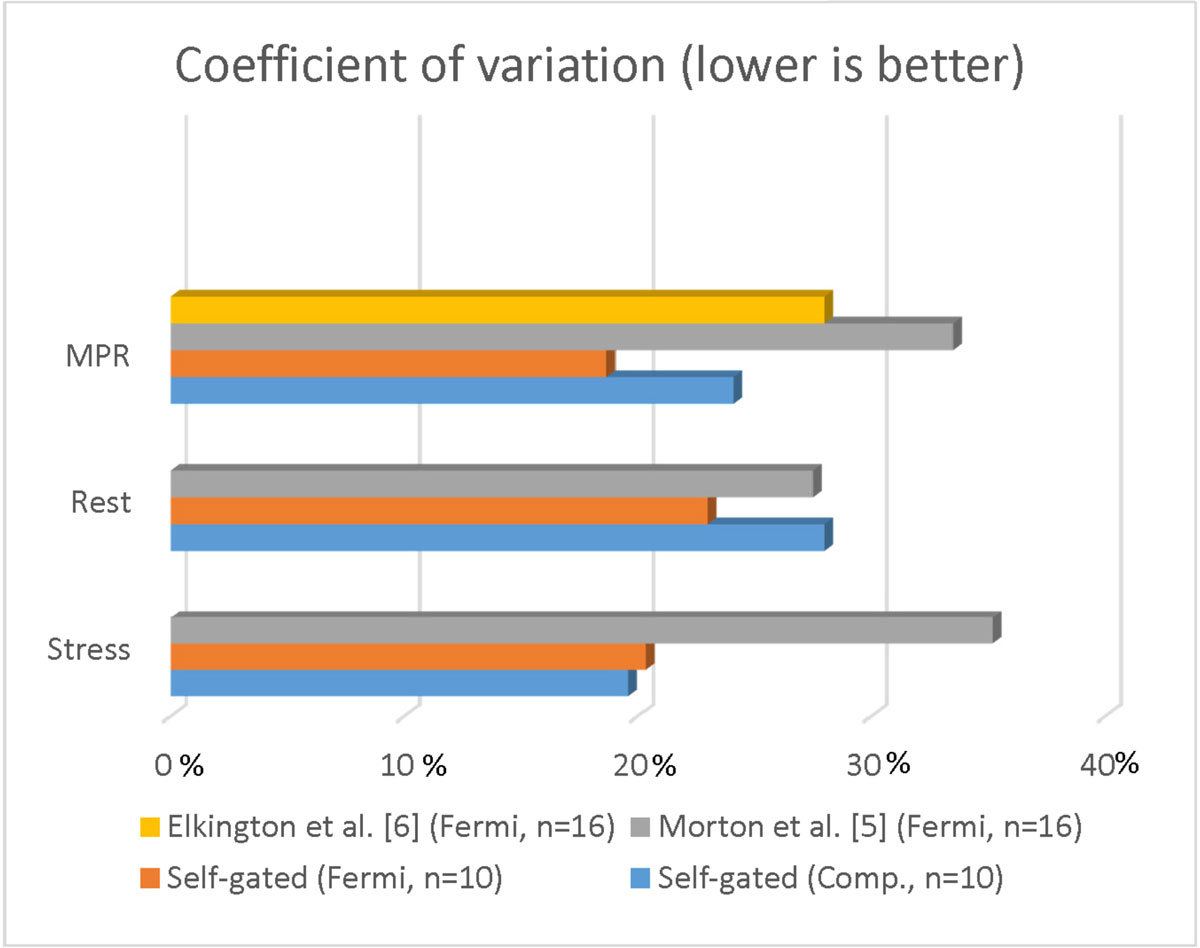
**Bar graph comparing the CoV between the self-gated systole and the published studies**.

Figure 1 compares the MPR estimates using self-gated systole datasets between scan 1 and scan 2. Figure 2 shows the estimates of coefficient of variation (CoV) obtained using the self-gated CMR compared to published ECG-gated studies. The published ECG-gated studies used the Fermi model for the estimation of MBF.

## Conclusions

The repeatability of the multi-slice self-gated systole CMR as measured by CoV [5,6] using both the Fermi and compartment model was similar or better than published single slice gated studies [5, 6]. Although the repeatability of MPR using the Fermi model was better than the compartment model, this was not statistically significant (p=0.27).
